# IGFBP6 Modulates Proteostasis by Activating ATF4 Targets and Reducing ER Retrotranslocon Expression

**DOI:** 10.1134/S1607672924600714

**Published:** 2024-10-31

**Authors:** O. E. Kolodeeva, O. E. Kolodeeva, I. D. Antipenko, A. A. Fatkulin, M. R. Yakhina, J. A. Makarova

**Affiliations:** Faculty of Biology and Biotechnology, National Research University Higher School of Economics, Moscow, Russia

**Keywords:** viscumin, ER, MDA-MB-231, *IGFBP6*, UPR, ERAD

## Abstract

Reduced expression of the IGFBP6 protein leads to an increase in the metastatic potential of breast cancer (BC) cells. The level of protein synthesis in tumor cells is increased, leading to a compensatory adjustment of proteostasis. One of the tools used to study proteostasis is protein toxins of the RIP-II family, which irreversibly inactivate ribosomes (particularly, viscumin). We investigated the effect of *IGFBP6* gene knockdown on the proteostasis in the BC cell line MDA-MB-231. Ribosomes from MDA-MB-231^IGFBP6^ cells, knockdown for the *IGFBP6* gene, are less efficiently modified by the toxin. This is probably due to the reduced transport of the viscumin catalytic subunit from the ER to the cytoplasm. MDA-MB-231^IGFBP6^ cells showed reduced expression of the retrotranslocon HRD1/Derlin subunit, which is a component of the ER-associated protein degradation system (ERAD). For ATF4 transcription factor, which is a part of the ER unfolded protein response (UPR) pathway, an increased expression of its targets was found.

## INTRODUCTION

An effective cellular response to the accumulation of unfolded proteins is essential for maintaining proteostasis. It is mediated by the unfolded protein response (UPR) system and the ER-associated protein degradation (ERAD) system. Three UPR sensors—PERK, ATF6, and IRE1α— are located in the ER membrane. Each of them governs regulatory cascades, with ERAD being activated only by ATF6 and IRE1α [1].

The cytotoxic proteins of the RIP-II family, one of which is ricin, serve as a tool for studying UPR and ERAD. These proteins penetrate the cytoplasm from the cell surface. By means of retrograde transport, they first enter the ER and then the cytoplasm. In the ER, the disulfide bond between two toxin subunits is reduced, after which one of them (the A-subunit) is transported into the cytoplasm using the ERAD system [2, 3]. Ricin has provided some important insights into the functioning of ERAD; however, its high toxicity hampers more detailed studies. A related but less toxic protein, viscumin (Mistletoe lectin I, ML-I), which is contained in mistletoe (*Viscum album*) and can inhibit protein synthesis in the cell, may be a good alternative [4]. In large doses, it leads to death; however, in small doses it can have a therapeutic effect. Therefore, viscumin is considered as an antitumor drug. The main mechanism of its action, similarly to ricin, is the hydrolysis of the glycosidic bond A-4324 in 28S rRNA, which leads to termination of protein synthesis [5].

Previously, it was shown that the MDA-MB-231 breast cancer cells knockdown for *IGFBP6*, MDA-MB-231^IGFBP6^, has an increased level of proliferation and higher metastatic potential compared to control [6, 7]. IGFBP6 binds the growth factors IGF1 and IGF2. The mechanism of its antioncogenic action remains obscure. It is known that, due to active proliferation, cancer cells usually have a high level of protein synthesis and therefore require correction of proteostasis. This is often achieved by increasing the UPR intensity.

In this work, we studied the effect of viscumin on MDA-MB-231 breast cancer cells with *IGFBP6* gene knockdown. We showed a higher resistance of their ribosomes to the toxin, which may be due to reduced expression of ERAD components. We found activation of expression of targets of the transcription factor (TF) ATF4, which is part of the UPR PERK-eIF2α-ATF4 pathway.

## MATERIALS AND METHODS

### Cell Lines

MDA-MB-231 cells were transfected with lentiviral vectors pLVX-shRNA1 (Clontech Laboratories, United States) with shRNA to the *IGFBP6* gene (MDA-MB-231^IGFBP6^ cell line) and control shRNA to the luciferase gene of the firefly *Photinus pyralis* (MDA-MB-231^luc^ cell line) [8]. MDA-MB-231^luc^ and MDA-MB-231^IGFBP6^ cells were cultured in 25-cm^2^ culture flasks (Corning, United States) at 37°C with 5% CO_2_ in DMEM/F12 medium (Gibco, United States) supplemented with penicillin and streptomycin (PanEco, Russia) to a final concentration of 100 U/mL and 100 μg/mL, respectively (PanEco, Russia), 10% FBS (HyClone, United States), and 1% GlutaMAX^TM^ (Gibco, United States).

### Estimation of the Proportion of Viscumin-Modified Ribosomes

The proportion of modified ribosomes was determined by real-time PCR. The primers to 28S rRNA corresponded to: (1) the region with an unmodified nucleotide (RIPII_nmod), (2) the region with a modified nucleotide (RIPII_mod), and (3) the region remote from the depurinated site for detection of all 28S rRNA transcripts (RIPII_ctrl) (Table 1). Vilo-revertase, after reaching the depurinated site, incorporates dATP into cDNA, whereas in an intact site it incorporates dTTP. Based on this feature, we detected the modified and unmodified regions of 28S rRNA. The PCR cycle threshold values (Ct) for RIPII_ctrl were used to normalize the values obtained for RIPII_mod and RIPII_nmod in each RNA sample.

**Table 1.** Primer sequences and efficiency

Gene	Primer	Efficiency	PCR product length, bp
*HERPUD1*	f-5'-ACCCCAACAATAACTTACAGGAAGG r-5'-ATAAAGGAGGGGCTGGTCTGC	1.99 ± 0.07	109
*HERPUD2*	f-5'-GGTCATCAGCAGGCTCCCAA r-5'-CCATCATCCATAAGACGCTCCATTT	2.06 ± 0.07	101
*SEL1L*	f-5'-ACCAGCTTTGACCGCCATTG r-5'-GCAGCCTCTTCTTCAGTTTCACAA	2.07 ± 0.08	189
*EDEM2*	f-5'-TATCGGGCTGGTCGGCAA r-5'-CCAGTCATCGAAGCGGGTGT	1.89 ± 0.08	196
*AUP1*	f-5'-TCAGTCCCTACCCACAGCCT r-5'-CGTCTCTCTGTGAATCTCCTTCTTG	2.13 ± 0.12	168
*SYVN1*	f-5'-GAGGACCGTGTGGACTTTATGGA r-5'-GGATGCTGTGATAGGCGTGG	1.97 ± 0.09	130
*DERL1*	f-5'-CCACACCTCAGTTTTTGTACCGC r-5'-AGTTGTGTCTCCCGCCTCC	1.99 ± 0.06	123
*DERL2*	f-5'-AGGCAGGCAGTTACAGGGTT r-5'-CTGTCAAGCAACACAGGGCT	2.15 ± 0.08	178
*DERL3*	f-5'-TTACACCGCAGCCTGTGTCC r-5'-CGGCAGTAGCGGAACACGA	1.93 ± 0.19	192
*SEC61A1*	f-5'-GTGGTCATCTATTTCCAGGGCTTC r-5'-TGCAGGATGATGGGGATGTTGG	2.17 ± 0.04	122
*SEC61A2*	f-5'-AAGGGTTACGGCTTGGGGTC r-5'-CCCTCAAACTCAGTACCTCTGCC	1.93 ± 0.09	122
*SEC61B*	f-5'-CACCCTCATCTCCAATATGCCTG r-5'-GCACTCCTTGTCCCACAGC	2.02 ± 0.08	147
*SEC61G*	f-5'-AAGGACTCCATTCGGCTGGTT r-5'-CACCCTCACACTTGTTCACCAAT	2.05 ± 0.04	218
*RIPII_nmod*	f-5'-TGCCATGGTAATCCTGCTCAGTA r-5'-TCTGAACCTGCGGTTCCTCT	1.87 ± 0.15	45
*RIPII_mod*	f-5'-TGCCATGGTAATCCTGCTCAGTA r-5'-TCTGAACCTGCGGTTCCACA	2.0 ± 0.2	45
*RIPII_ctrl*	f-5'-GATGTCGGCTCTTCCTATCATTGT r-5'-CCAGCTCACGTTCCCTATTAGTG	2.05 ± 0.2	81

### RNA Extraction

MDA-MB-231^luc^ and MDA-MB-231^IGFBP6^ cells were lysed using QIAzol Lysis Reagent (QIAGEN, Germany). RNA was extracted using the miRNeasy Micro Kit (50) (QIAGEN, Germany) according to the manufacturer’s protocol. The concentration and purity of RNA samples were measured using a NanoDrop ND-1000 spectrophotometer (Thermo Fisher Scientific, United States).

### Reverse Transcription and Quantitative RT-PCR

Reverse transcription was performed using the SuperScript VILO cDNA Synthesis Kit (Thermo Fisher Scientific, United States); 200 ng of RNA were used in the reaction. The reaction was performed in a DNA Engine Tetrad 2 Peltier Thermal Cycler (Bio-Rad, United States). Quantitative real-time PCR (qRT-PCR) was performed using the 5X qPCRmix-HS SYBR Kit (Eurogen, Russia). The sequences of the primers used are given in Table 1. The primers were selected using the Primer-BLAST software. The ability of the primers to form secondary structures (hairpins), homodimers, and heterodimers was assessed using the OligoAnalyzer 3.1 software. The melting temperature of the primers was 64 ± 2°C. The final concentration of the primers was 0.25 μM. cDNA diluted 15 times with nuclease-free water was added to the reaction mixture to measure the expression of the target genes or cDNA diluted 50000 times was added to estimate the proportion of inactivated ribosomes. Amplification was performed using a DTprime detecting amplifier (DNA-Technology, Russia). A total of 35 qPCR-RT cycles were performed (at 94°C for 20 s, at 64°C for 10 s, and at 72°C for 15 s).

### Bioinformatic Analysis of mRNA and Protein Expression

For analysis of transcriptomes of MDA-MB-231^luc^ and MDA-MB-231^IGFBP6^ cells, the original sequencing data obtained by us earlier (GSE247735) were used [8]. Raw reads were preprocessed using fastp 0.23.2 and then mapped to the human reference genome (GRCh38) using STAR 2.7.10b. Differential gene expression analysis was performed using the R package DESeq2. Correction for multiple hypothesis testing was performed using the Benjamini–Hochberg method. Genes with an adjusted *p* value (false discovery rate, FDR) < 0.05 were considered in the analysis. The median of ratios normalization from the R package DESeq2 was independently applied to the original matrix, and the obtained values were normalized to gene lengths (kb), which made it possible to compare expression levels both between samples and between genes within a single sample. For proteome analysis of MDA-MB-231^luc^ and MDA-MB-231^IGFBP6^ cells, we used normalized log2 iBAQ values [6].

To analyze the representation of activated targets of transcription factors in the MDA-MB-231^IGFBP6^ line, the TRRUST v2 database (https://www.grnpedia.org/trrust) was used. Only the activatory interactions between TFs and targets were considered. To analyze the significance of the number of activated targets of each TF, a one-sided Fisher’s exact test (fisher_exact, SciPy library 1.13.1) was used. The target was considered activated if there was a change in expression in the knockdown line by more than 1.5 times (FDR < 0.05) compared to the control line. Enrichment of activated targets of TFs was considered to be observed if the FDR value of the Fisher’s exact test was less than 0.05.

## RESULTS AND DISCUSSION

### IGFBP6 Knockdown Reduces the Efficiency of Ribosome Modification by Viscumin

After the treatment with viscumin, the number of damaged ribosomes in MDA-MB-231^IGFBP6^ cells was lower than in control MDA-MB-231^luc^ cells; additional incubation for 24 h in a viscumin-free medium increased this difference (Table 2). This could be due to less efficient transport into the cytoplasm and/or more efficient degradation of viscumin by MDA-MB-231^IGFBP6^ cells.

**Table 2.** The percentage of modified ribosomes in MDA-MB-231^luc^ and MDA-MB-231^IGFBP6^ cells during cell treatment with viscumin (100 nM) for 6 h and for 6 h followed by 24 h incubation (in triplicate)

Incubation time, h	MDA-MB-231^luc^, %	MDA-MB-231^IGFBP6^, %
0 (Control)	0 ± 0	0 ± 0
6	20.1 ± 2.8	16.8 ± 1.1
6 + 24	41.5 ± 5.8	25.2 ± 1

Viscumin, similarly to other toxins of the RIP-II family, enters the cytoplasm from the ER via the ERAD system. The transport of ER lumen proteins to the cytoplasm is mediated by a retrotranslocon consisting of the E3-ubiquitin ligase HRD1, one of three proteins of the derlin family (DERL1-3), one of the proteins HERPUD1 and HERPUD2, as well as the adaptor protein SEL1L [1]. This retrotranslocon is also involved in the transport of ricin [3]. The transport of ricin is also mediated by the ER translocon Sec61 [9], which is also involved in ERAD [10].

### Knockdown of IGFBP6 Alters the Expression of Some Genes Involved in the Transport and Degradation of ERAD Substrates

**Fig. 1.**
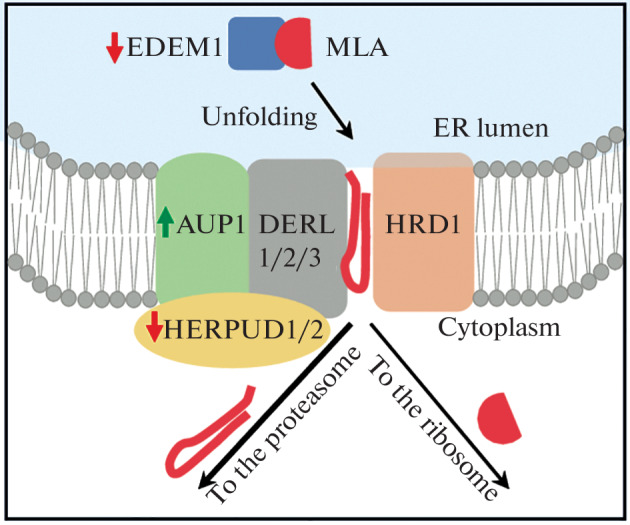
A decrease in EDEM2 and HERPUD1/2 retrotranslocon component expression can reduce the transport of viscumin from the ER to the cytoplasm, and an increase in AUP1 expression can lead to increased ubiquitination and degradation of viscumin. MLA is viscumin A-subunit.

Analysis of transcriptomes of MDA-MB-231^luc^ and MDA-MB-231^IGFBP6^ cells showed that the expression of the HERPUD1 and HERPUD2 retrotranslocon components in MDA-MB-231^IGFBP6^ cells is reduced compared to the control. A decrease in HERPUD1 expression is also shown using qRT-PCR, and a tendency towards a decrease in HERPUD2 expression is detected in the proteomes (Table 3). Both proteins are required for the proper organization and functioning of the retrotranslocon [1]. The expression of SEL1L and, in addition, the ER resident lectin EDEM2, which initiates ERAD of lumen proteins, performing the first step in the elimination of carbohydrate residues from soluble ERAD substrates, is also reduced [11]. EDEM2 targets soluble ERAD substrates as well as ricin to the translocon [12]. The described changes may contribute to a decrease in the transport of ERAD substrates (including probably viscumin, which is related to ricin) from the ER to the cytoplasm (Fig. 1). Reduced DERL3 expression probably does not affect the intensity of transport, because the gene is practically not expressed in both lines (Table 3). It should be noted that all proteins of the HRD1/Derlin retrotranslocon were shown to be involved in ricin transport in a genome-wide screening using shRNA [13], or in separate experiments [3] (Table 3).

**Table 3.** Expression of components of the HRD1/Derlin retrotranslocon, the Sec61 translocon and some auxiliary elements that transport soluble proteins between the ER lumen and the cytoplasm. Data from the analysis of the transcriptomes and proteomes of MDA-MB-231^luc\IGFBP6^ and qRT-PCR are shown. The fold change is presented in a linear scale. “–"—The fold change is less than 1.1. N.d.—protein is not found in the proteome. All fold change values in the transcriptome have FDR adjusted *p*-value < 0.001. Genes with significant coordinated expression changes according to both transcriptomic and qRT-PCR data and with relative expression in transcriptomes > 1.0 are highlighted in bold.

Gene name	Transcriptome, relative expression		Fold change	qRT-PCR		Proteome		Reduced expression protects against ricin
	MDA-MB-231^luc^	MDA-MB-231^IGFBP6^		fold change	FDR	fold change	FDR	
*HRD1/Derlin retrotranslocon and associated proteins*								
*HERPUD1*	93.6	66.0	**–1.4**	**–1.6**	3e–3	N.d.		+ [13]
*HERPUD2*	70.3	54.2	–1.3	–1.2	0.20	–2.0	0.59	+ [13]
*SEL1L*	134.1	1069	–1.3	1.2	0.19	–1.1	0.90	+ [13]
*HRD1* *(SYVN1)*	125.6	125.4	–	–1.3	0.09	N.d.		–
*DERL1*	79.3	72.5	–1.1	–1.1	0.59	–1.8	0.38	+ [13]
*DERL2*	71.7	67.7	–1.1	1.1	0.85	1.9	0.34	+ [13]
*DERL3*	0.7	0.4	–1.6	–5.5	9e–7	N.d.		+ [13]
*EDEM2*	84.2	50.0	**–1.7**	**–1.5**	0.01	N.d.		+ [3]
*AUP1*	317.0	621.6	**2.0**	**1.5**	0.01	**2.4**	0.02	**–**
*Sec61 translocon*								
*SEC61A1*	391.2	402.7	**–**	**–**	0.86	1.3	0.25	**–**
*SEC61A2*	35.3	43.8	1.2	–2.2	3e–3	N.d.		**–**
*SEC61B*	255.1	188.7	**–1.4**	**–1.8**	0.04	1.1	0.13	**–**
*SEC61G*	84.7	109.5	1.3	–	0.87	1.1	0.25	**–**

In addition, both transcriptome analysis and qRT-PCR revealed a decrease in the expression of the SEC61B subunit of the Sec61 translocon (Table 3). The decrease in the SEC61B expression may contribute to the reduced accessibility of MDA-MB-231^IGFBP6^ ribosomes to viscumin.

At the same time, all three methods of analysis showed an increased expression of the *AUP1* gene (Table 3), which is involved in the ubiquitination of ERAD substrates translocated into the cytoplasm via the HRD1/Derlin translocon [1]. This may contribute to a more intense degradation of ERAD substrates, including viscumin, by MDA-MB-231^IGFBP6^ cells (Fig. 1). The described changes indicate alterations in  the ERAD system and may contribute to the lower   availability of ribosomes for viscumin in MDA-MB-231^IGFBP6^ cells.

### IGFBP6 Knockdown Activates Targets of ATF4, an UPR-Mediating TF

For all TFs, the changes in expression of their target genes were analyzed using the hypergeometric test. Table 4 presents the TFs whose activated targets are in the MDA-MB-231^IGFBP6^ cells are significantly overrepresented (target gene expression increases more than 1.5-fold, FDR < 0.05). The largest proportion of such targets was found for ATF4, a TF activated by the resident ER sensor PERK, which triggers UPR under ER stress.

**Table 4.** TFs with the highest percentage of activated targets

TF	Odds ratio	Number of activated targets/number of all targets	*p*-value	FDR
ATF4	6.10	8/21	3e–4	0.04
JUN	4.20	19/64	3e–6	1e–3
MYC	3.82	15/54	7e–5	0.01
RELA	2.43	31/158	4e–5	0.01

In our laboratory, it was previously shown that *IGFBP6* knockdown enhances proliferation and metastatic potential of MDA-MB-231 cells [6]. Cancer cells, due to increased proliferation, have a high level of protein synthesis, which creates a load on the folding mechanisms. Therefore, in many types of cancer, including breast cancer, a compensatory activation of UPR is observed, which allows reconfiguring proteostasis to the needs of the cancer cell [14, 15]. ATF4 activation in MDA-MB-231^IGFBP6^ cells may contribute to such UPR activation.

Three other TFs (Table 4) are transcription activators that enhance cell proliferation. Their activation at a reduced IGFBP6 expression may contribute to the enhancement of the oncogenic phenotype of MDA-MB-231^IGFBP6^ cells.

In addition, both Jun and Myc are involved in the complex regulation of UPR. Jun is activated in response to UPR and, in turn, activates UPR by binding to the promoters of genes involved in it, including the ATF4 promoter [16]. Myc also activates UPR genes by binding, in particular, to the ATF4 promoter, and ATF4 stabilizes Myc, preventing its proteasomal degradation [17]. Rela is a subunit of NFKB, which is activated during the UPR, including through the PERK–ATF4 pathway [18].

Interestingly, despite the increase in UPR activity, no increase in ERAD was detected. This may be due to the fact that ERAD is activated by the IRE1α and ATF6 sensors (whose expression which did not change), rather than by PERK-ATF4.

Thus, as a result of viscumin treatment, a smaller number of ribosomes are inactivated in MDA-MB-231^IGFBP6^ breast cancer cells compared to the control. This may be caused by a decreased transport of the toxin from the ER to the cytoplasm, due to decreased expression of the ERAD system components, as well as its more efficient proteasomal degradation. Increased expression of ATF4 TF targets indicates activation of UPR. Changes in expression of ERAD and UPR components indicate alterations in proteostasis as a result of decreased *IGFBP6* expression. The negative correlation of UPR activity with the *IGFBP6* expression level may contribute to the antioncogenic effect of this gene.

## ABBREVIATIONS AND NOTATION

ER: endoplasmic reticulum;
UPR: unfolded protein response;
ERAD: ER-associated degradation;
RIP-II: ribosome-inactivating proteins type II;
BC: breast cancer;
TF: transcription factor.
